# Discovering microbe-disease associations from the literature using a hierarchical long short-term memory network and an ensemble parser model

**DOI:** 10.1038/s41598-021-83966-8

**Published:** 2021-02-24

**Authors:** Yesol Park, Joohong Lee, Heesang Moon, Yong Suk Choi, Mina Rho

**Affiliations:** 1 Department of Computer Science and Engineering, Hanyang University, Seoul, Korea; 2 Department of Biomedical Informatics, Hanyang University, Seoul, Korea

**Keywords:** Data mining, Literature mining, Machine learning, Computational biology and bioinformatics, Microbiology

## Abstract

With recent advances in biotechnology and sequencing technology, the microbial community has been intensively studied and discovered to be associated with many chronic as well as acute diseases. Even though a tremendous number of studies describing the association between microbes and diseases have been published, text mining methods that focus on such associations have been rarely studied. We propose a framework that combines machine learning and natural language processing methods to analyze the association between microbes and diseases. A hierarchical long short-term memory network was used to detect sentences that describe the association. For the sentences determined, two different parse tree-based search methods were combined to find the relation-describing word. The ensemble model of constituency parsing for structural pattern matching and dependency-based relation extraction improved the prediction accuracy. By combining deep learning and parse tree-based extractions, our proposed framework could extract the microbe-disease association with higher accuracy. The evaluation results showed that our system achieved an F-score of 0.8764 and 0.8524 in binary decisions and extracting relation words, respectively. As a case study, we performed a large-scale analysis of the association between microbes and diseases. Additionally, a set of common microbes shared by multiple diseases were also identified in this study. This study could provide valuable information for the major microbes that were studied for a specific disease. The code and data are available at https://github.com/DMnBI/mdi_predictor.

## Introduction

With recent advances in biotechnology and sequencing technology, beneficial and deleterious effects of bacterial composition in humans and animals have been rigorously investigated. In particular, large-scale studies have extensively investigated the microbial composition associated with a specific disease^1–3^. Several studies have reported the effects of diverse microbes on various diseases^4–6^, including cancer^7^, vascular disease^8^, and autoinflammatory disease^9^. Critical bacterial infections cause serious problems and even death^10^. Determining the role or the correlation of microbes in the development of a disease is very important to understand disease pathology and diagnosis markers.

Several studies have provided databases of the curated taxonomic information or sequencing resources related with microbes and diseases. For example, Human Microbe-Disease Association Database (HMDAD) has 483 microbe-disease associations manually curated from 61 previously published articles^11^. Human Pan-Microbe Communities Database (HPMCD) provides over 1800 curated human gastrointestinal metagenome resources^12^. gutMDisorder provides microbe-related disorder and intervention information that were extracted from scientific articles^13^. Even though these databases are valuable resources for analyzing diseases-related microbial information, such information was extracted from a limited number of publications. In order to use wide resources publicly available as scientific articles more systematically and comprehensively, efficient text mining methods need to be developed. Recently developed computational methods predict the association between microbes and diseases^14–19^. Such predictions are made from the pre-defined microbe-disease association networks by using various graph algorithms and kernel functions. For example, KATZHMDA applied KATZ measure to calculate the potential similarity between microbes and diseases using a microbe-disease association network^14^. Several variations were also introduced by using a depth-first search, neighbor-based collaborative filtering, Laplacian regularized least squares, bidirectional label propagation, and bi-random walk^15–19^.

The number of published biomedical articles increases at an exponential rate, and extracting information from such a large-scale collection of literature requires a high cost. Efficient text mining methods have emerged to address this problem. Methods have been developed using named entity recognition (NER), normalization of the entities, relation extraction, and relation classification^20–24^. The NER and normalization of entities are important preprocessing steps for extracting relational information. In recent years, machine learning approaches such as conditional random field and neural networks have been dominant^20–25^. For example, BANNER^20^ is a trainable biomedical named entity recognition system based on conditional random fields^26^. Recurrent neural networks (RNN) have shown good performance with natural language processing, and long short-term memory (LSTM) was developed to add cell states to the RNN, which improved vanishing gradient problems^27^. DNorm^22^ is a system used for normalizing disease names in biomedical texts by learning the similarities between mentions and concept names based on pairwise learning to rank^28^. Collections of biomedical terms such as gene ontology (GO)^29^, BioThesaurus^30^, unified medical language system^31^, medical subject headings (MeSH) terms^32^, and the Comparative Toxicogenomics Database^33^ have been used to solve this problem. Resources such as the NCBI disease corpus^34^ and BioCreative V CDR corpus (BCVCDR)^35^ have been used as the gold standard in training and testing data for NER and normalization.

To extract or classify relationships between biomedical entities, rule-based decision, pattern matching, or machine learning have been explored^25,36–46^. RelEx is a method for predicting interactions by applying rules to dependency parse trees, focusing on the relationship between genes and proteins^36^. @MInter predicts interactions between microorganisms using support vector machines and builds a database^37^. Protein–protein interaction and drug–drug interaction (DDI) have been explored in the biomedical literature to identify positive and negative influences between proteins and between drugs, respectively^25,38–42^. For extraction of such relations, two different types of results are expected. First, the relation between entities is directly detected, and second, such a relation is classified into predefined classes, such as ADVICE, EFFECT, INT, and MECHANISM in DDI interaction. The relationship between diseases and genes has also been explored^25,43,44^. The microbial phenotypic traits and other associations that were obtained from the literature have been investigated by network analysis^46^.

Although a tremendous amount of literature related to microbes and diseases is available, text mining methods that focus on the relation between microbes and diseases have been rarely studied to date. In this study, we have developed a method that extracts the microbe-disease relationship from the biomedical literature by combining natural language processing (NLP) and machine learning methods. Our NER and normalization methods for microbes and diseases were applied in the pre-processing. A variant of RNN was constructed to obtain sentences that contain a microbe-disease relationship. Subsequently, the relation words were predicted from the retained sentences by combining the results from two different parsing methods. As a case study, a large-scale microbe-disease relation network analysis was performed to provide valuable information on whether a set of specific microbes are common or exclusive to a given disease or not. Since the proposed method provides a systematic way of extracting the microbe-disease relations with high accuracy from scientific literature, it can be a useful resource for studying the microbial involvement in disease development and pathophysiology in a comprehensive manner. Considering the massive size of scientific literature, the current databases of microbe-disease relations contain only a limited number of publications. Therefore, our large-scale text analysis approach could provide more detailed information of the microbe-disease relation.

## Materials and methods

**Figure 1:**
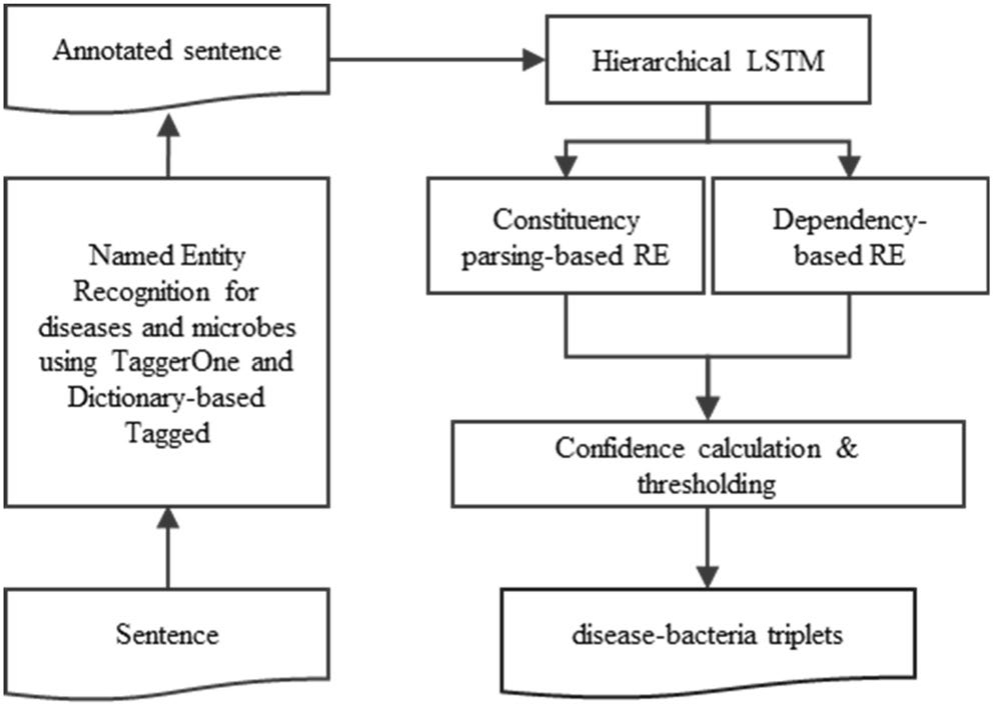
Workflow of extracting the associations between microbes and diseases. The NER process uses two approaches: a semi-Markov model for disease and a dictionary-based method for bacteria, bacterial strain, and virus. Relation is determined using a hierarchical LSTM, and the relation word was extracted by an ensemble model of constituency parsing-based and dependency-based methods.

The proposed system consists of three steps: (1) NER that annotates the terminology for microbe and disease using a dictionary-based method and a semi-Markov model; (2) binary classification for relation detection using a hierarchical LSTM model; and (3) an ensemble method for relation extraction, which uses constituency parsing-based structural pattern matching and dependency-based relation extraction (Fig. 1). In the ensemble method, the confidence scores were calculated to extract relations more accurately by complementing two different approaches.

### Collection of biomedical corpus and named entity recognition

To evaluate the performance of relation detection, two different data sets were used in this study. The first data set is a golden standard for drug–drug interaction DDIE2013^47,48^. The second data set of microbe-disease association is generated in-house. A total of 1100 random sentences that contain the names of both the disease and the microbe were obtained from PubMed abstracts. The relation between two entities of microbe and disease was manually annotated by domain experts. If one or more relations were found between entities in the sentence, each pair was annotated. Among the words that describe the relation, a more specific word was regarded as the relation word. For instance, in the sentence "BAC00Pseudomonas_aeruginosa is a pathogen that frequently causes DIS00acute_lung_injury.", the word *causes* was regarded as the relation word. Among 1100 sentences, 1000 were used for training, and 100 sentences were used for testing.

The entities of microbe and disease were recognized independently from the sentences. For disease, we performed NER and normalization using TaggerOne^24^, a machine learning tool that recognizes and normalizes multiple concept entities using a semi Markov model. TaggerOne divides the sentences into segments consisting of one or more tokens. It subsequently performs NER and normalization simultaneously by estimating the score for the segment as the sum of the NER score and the normalization score. We used two TaggerOne models, which were trained using NCBI and BCVCDR corpus, respectively. In order to avoid mis-annotation of disease names with bacteria and virus names, dictionary-based NER was also applied based on the NCBI taxonomy information. The bacterial names were extended to include specific strain information. The list of bacteria, bacterial strains, and a list of viruses were downloaded from the NCBI website.

### Relation detection with hierarchical long short-term memory

#### System overview for relation detection between entities

**Figure 2:**
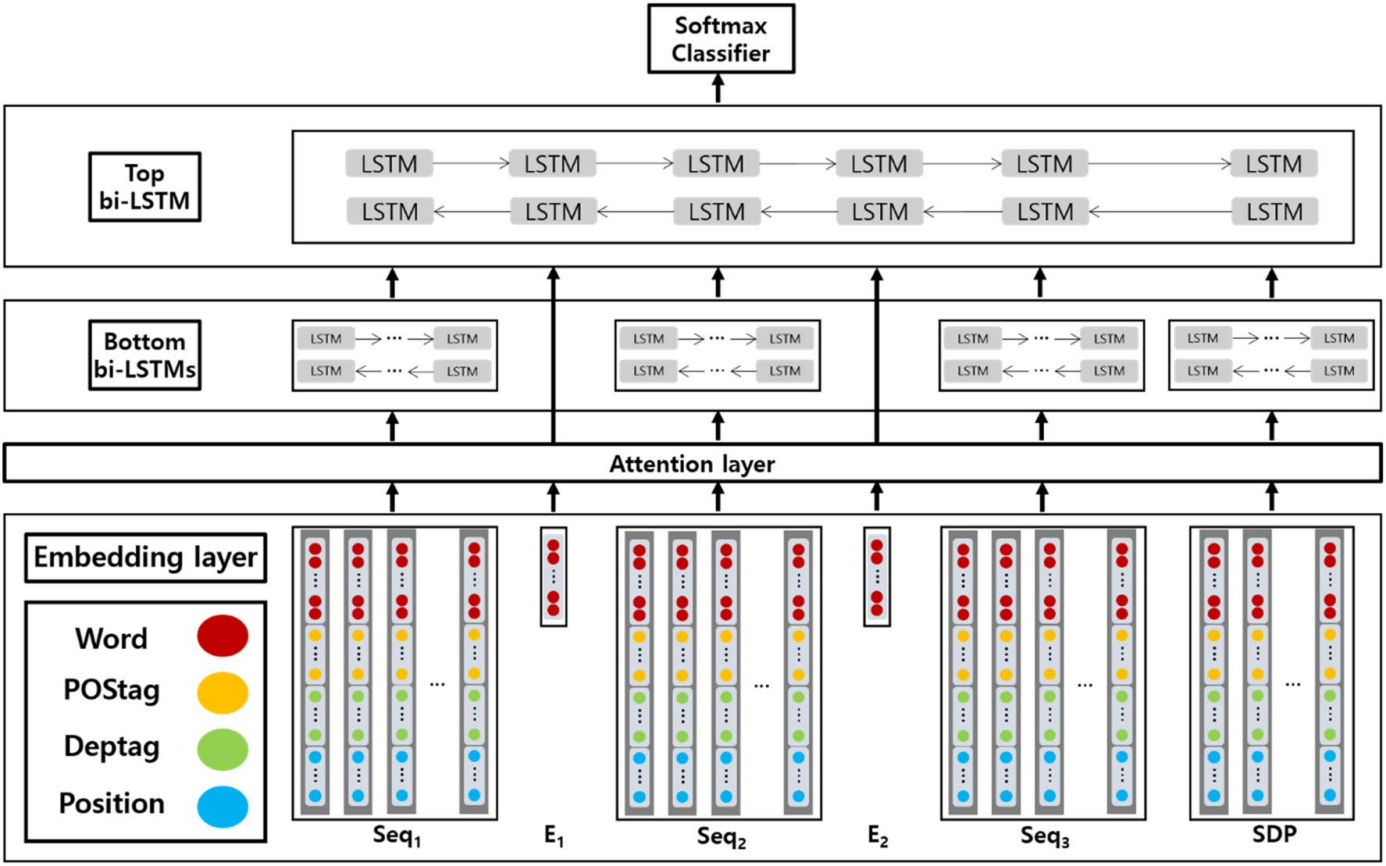
The overview of hierarchical long short-term memory model in this study. Words, part of speech for words, dependency tags for words, and relative positions to entities for words are used as features in the model. The model consists of embedding layer, attention layer, bottom LSTM, top LSTM, and softmax classifier. As input, the phrase before the first entity (${\mathrm{Seq}}_{1}$), the first entity (${\mathrm{E}}_{1}$), the phrase between two entity (${\mathrm{Seq}}_{2}$), the second entity (${\mathrm{E}}_{2}$), the phrase after the second entity (${\mathrm{Seq}}_{3}$), and shortest dependency path (SDP) are entered into the model.

We used hierarchical LSTM to determine the existence of the relationship between bacteria and disease. The LSTM was constructed hierarchically, considering the entities, which were adapted and improved from a previous study^49^. The hierarchical LSTM model consists of six layers: an input layer, embedding layer, attention layer, bottom LSTM layer, top LSTM layer, and output layer (Fig. 2). The input of the hierarchical LSTM includes a sentence and its shortest dependency path. A sentence contains two entities, which divides the sentence into three phrases: the words before the first entity, the words between two entities, and the words after the second entity. The shortest dependency path was obtained from the sentence by Stanford dependency parser to further consider contextual meaning.

#### Embedding layer

In the embedding layer, each sentence was divided into words that were vectorized. For word embedding, we used word2vec models^50^, which were retrained with a corpus from DDI^47,48^, and the bacteria-disease relation were generated in this study using biomedical scientific literature in PubMed and PMC^51^. The additional features for each word were obtained from part of speech (POS), dependency tag, and positions. POS and dependency tags were obtained by the Stanford dependency parser, and they could better express the word because POS and dependency tags of the same word were different depending on the sentence. For POS and dependency tag embedding, a word2vec model was applied. The position of each word was the relative distance from the word to the entities. Positions were represented by one-hot encoding depending on the distance. The vector size for word embedding, POS, dependency tag, and positions was 200, 10, 10, and 20, respectively.

#### Attention layer

In the attention layer, entity-based attention^52^ was used. In the entity-based attention, the weight of the word $w_{i}$ based on the entities $e_{1}$ and $e_{2}$ is defined as follows:

1 $$\theta_{wi}^{k} = \frac{{exp\left( {dot\left( {w_{i}^{word} , e_{k}^{word} } \right)} \right)}}{{\mathop \sum \nolimits_{j = 1}^{m} exp\left( {dot\left( {w_{j}^{word} , e_{k}^{word} } \right)} \right)}} \left( {k \in \left\{ {1,2} \right\}} \right)$$

2 $$\theta_{wi} = \frac{{\theta_{wi}^{1} + \theta_{wi}^{2} }}{2}$$

If a specific word is closer than another word for an entity in the embedding space, it is given more weight through the dot product. Since we classify sentences for entities, we control weights for the entities.

#### LSTM layers

The bottom LSTM layer consists of four LSTMs: three LSTMs for three phrases in a sentence that were divided by two entities, and an LSTM for the shortest dependency path. LSTMs for three fragments have fixed 60 time steps, and an LSTM for the shortest dependency path has fixed 12 time steps. Each LSTM has a hidden size of 100. In an example sentence, "additionally, in otherwise healthy people, vulnificus causes wound infection that can require amputation or lead to sepsis" two pairs of the relation between vulnificus and infection, and between vulnificus and sepsis were extracted. For a pair between vulnificus and infection, three phrases, "additionally, in otherwise healthy people", "causes wound", and "that can require amputation or lead to sepsis" were obtained. The shortest dependency path in the example sentence is “causes wound”. Each sentence is padded or cut by the time steps before placing the LSTMs. Each LSTM consists of many to one bidirectional LSTMs (bi-LSTM), and the final result of the bottom LSTM layer is a 4 × 200 matrix.

The top LSTM layer is a bi-LSTM, which consists of six time steps and has a hidden size of 100. Each entity was embedded as a 1 × 200 matrix, which was combined with the results of the bottom LSTM layer to form a 6 × 200 matrix to be an input in the top LSTM. The top LSTM outputs a vector of length 200, and this output passes through a feed-forward neural network. The feed-forward neural network finally outputs the binary class results using the softmax function.

### Constituency parsing-based structural pattern matching

In order to extract relation words, a parse tree-based structural pattern-matching method, TPEMatcher^53^, was adjusted to our problem. TPEMatcher uses tree pattern expression (TPE) as a search query to express the structural pattern of parse trees. It allows the use of regular expressions to reveal string patterns and can express grammatical patterns of parse trees. Furthermore, TPE extracts information from a large text corpus with very low computational complexity.

**Figure 3:**
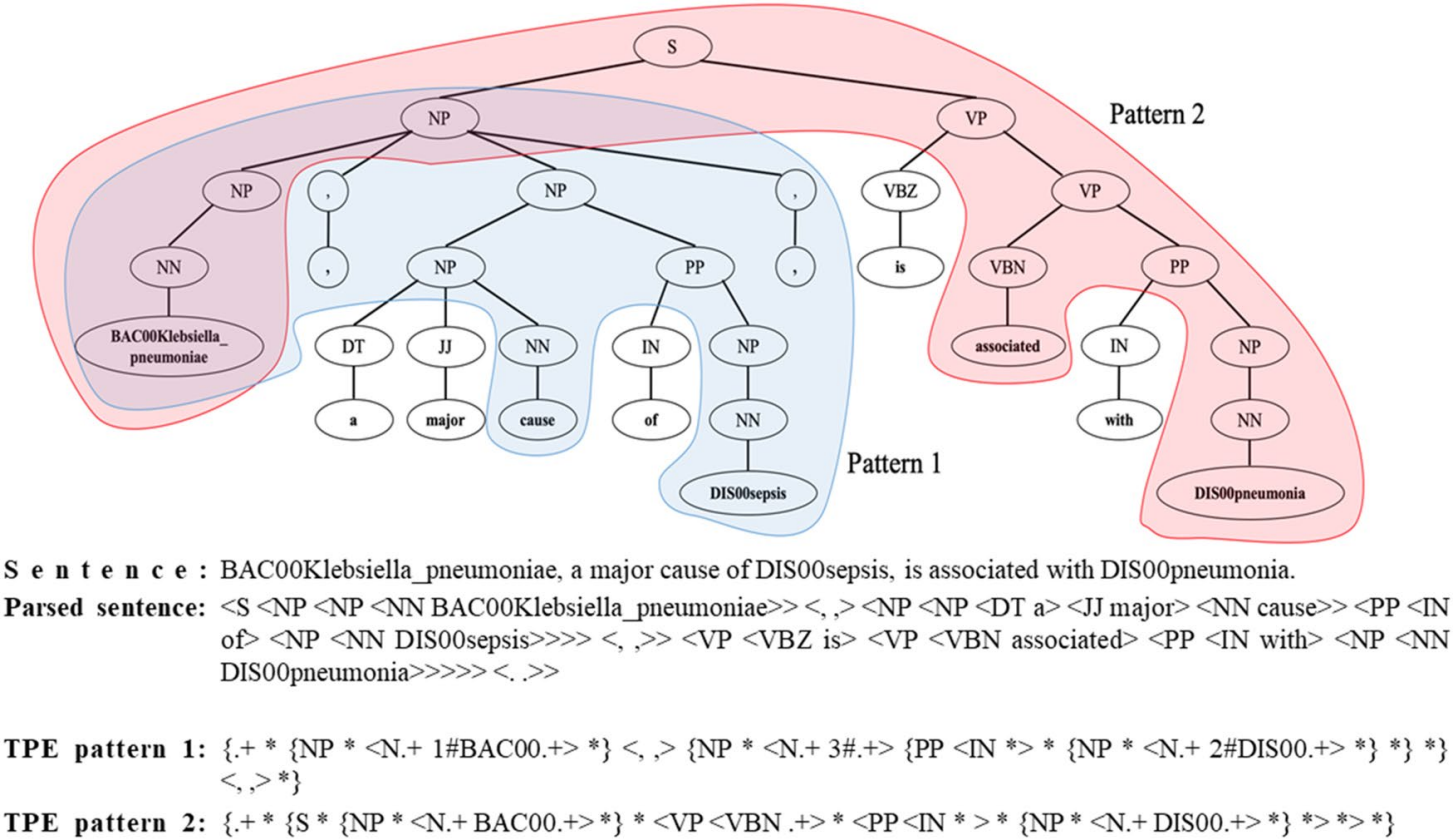
An example sentence processed by parse tree-based structural pattern matching. A given sentence is parsed to find the structural dependency. From the parsed sentence, two different tree pattern expressions, TPE pattern 1 and 2 were extracted from 59 predefined TPE patterns. The TPE pattern 1 (in blue) extracts the microbe-disease-relation triplet of (DIS00sepsis, BAC00Klebsiella_pneumoniae, cause). The TPE pattern 2 (in red) extracts the other triplet of (DIS00pneumonia, BAC00KLEbsiella_pneumoniae, associated).

TPE patterns are matched to each parse tree of a sentence in order to produce the matched parts of the parse tree as a search result. We constructed a set of 59 TPE patterns comprising microbes, diseases, and relation words from the corpus. For example, *the TPE pattern “{.*+ * {NP * < *N.* + *1#BAC00.* +> *} <*, ,*> {NP * < *N.* + *3#.* +> *{PP* < IN *> * {NP * < *N.*+ *2#DIS00.*+  > *} *} *} < *, ,* > *}” is one of these patterns to extract the relation triplets from the appositive phrase with commas (Fig. 3).

To parse sentences, the Stanford CoreNLP analyzer was applied. Each node of the TPE pattern was matched to a node or a subtree of the parse tree. Among the matched nodes of the parse tree, TPEMatcher extracted the words corresponding to microbes, diseases, and relations from nodes matched by “1#*BAC.*+”, “2#*DIS.*+”, and “3#*.*+”, respectively. In the case of TPE pattern 1, *BAC00Klebsiellapneumoniae*, *DIS00sepsis*, and *cause* were extracted, and then these words were stemmed and bundled into triplets (*BAC00Klebsiella_pneumoniae*, *DIS00sepsis*, *cause*) as the final output of TPEMatcher (Fig. 3). In addition, another triplet relation was found: (*BAC00Klebsiella_pneumoniae*, *DIS00pneumonia*, *associate*). To extract such a triplet, we crafted another TPE pattern “{S * {NP * <N.+ BAC00.+> *} * <VP <VBN .+> * <PP <IN *> * {NP * <N.+ DIS00.+> *} *> *> *}” for passive sentences with past participle (Fig. 3). As a final result, TPEMatcher extracted two triplets, (*BAC00Klebsiella_pneumoniae*, *DIS00sepsis*, *cause*) and (*BAC00Klebsiella_pneumoniae*, *DIS00pneumonia*, *associate*) from the given sentence.

### Dependency parsing-based relation extraction

In order to extract relations between microbes and diseases, dependency trees were built from the sentences using the Stanford CoreNLP library^54^. Since dependency parsing captures long-range syntactic relations, it can be complementary to the constituency parsing in relation extraction. Before the tree is traversed, three preprocessing steps to simplify the prediction procedure were performed: (1) chunking a group of words with the pattern of *word (of|with) entity* and a compound relation between the entity and its parent node; (2) excluding a pair of entities with the distance of more than 4 in the dependency tree (edges of *conj, conj:and, conj:or, compound,* or *appos* were not counted in the distance), and (3) extracting simple *effector-effected* relations that are connected by prepositions and relation words such as *by*, *in*, *from*, *on*, *with*, *of*, *due to*, *induced*, and *between*.

**Figure 4:**
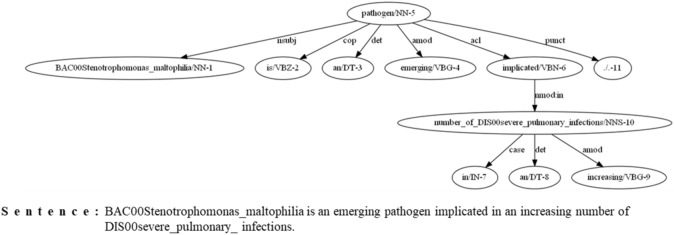
An example of microbe-disease relation extracted from a dependency tree. Two entity nodes, ‘BAC00Strenotrophomonas_maltophilia’ and ‘DIS00severe_pulmonary_infections’, have a lowest common ancestor of ‘pathogen’, which has a more descriptive child node of 'implicated' without a descriptive child node. Therefore, 'implicated' is extracted as the relation word between two entities of microbe and disease.

In the dependency tree, the subtree with a root of the lowest common ancestor (LCA) node between the two entities has essential information for the relation between the entities. In addition to the LCA node, more descriptive relation word can exist in the child node of LCA. If the LCA node has a child node that is connected by the edges such as *acl, acl:relcl, amod, xcomp, ccomp, appos, nmod:as, conj:and, conj:or, advcl,* and *dep*, the child is assigned as the relation word. For example, the relation word *implicated* was observed from our algorithm in a given sentence *“BAC00Stenotrophomonas _maltophilia is an emerging pathogen implicated in an increasing number of DIS00severe_pulmonary _infections.”* (Fig. 4). When one of the two entity nodes is LCA, the relation between the entities is extracted from the edge. The candidate pair might not have an LCA node. When the edge is not a preposition, the word combined with the entity as a chunk, is a relation.

When more than two words of the same entity type are connected, this phrase is represented as ancestor–descendant nodes in the tree. For example, there is an annotated sentence *“BAC00Streptococcus _pneumoniae, the pneumococcus, is the most common_cause of DIS00sepsis and DIS00meningitis.”* The entity *DIS00meningitis* has the parent node of the same type, which is *DIS00sepsis*. When *DIS00sepsis* has a *common_cause* relationship with *BAC00Streptococcus_pneumoniae*, a relation between *BAC00Streptococcus_pneumoniae* and *DIS00meningitis* is also *common_cause* because it inherits the relationship from the parent node.

### Ensemble model to combine relations

To combine the results from two complementary approaches of relation extraction, an ensemble model was applied. We assumed that the correctness of an extracted relation triplet is highly dependent on its relation word and extraction patterns in each module. Thus, we define the confidence scores based on Bayes’ theorem to determine the reliability of an extracted relation. The confidence of a relation triplet is determined by the maximum likelihood of patterns that extract the triplet as follows:

3 $${\text{conf}}\left( {r_{j} } \right) = \mathop {\max }\limits_{i} {\text{ Pr}}(p_{i} {|}r_{j} {)}$$

where $r_{j}$ is an extracted relation triplet that contains a relation word *j*, and $p_{i}$ is the *i-*th pattern that extracts the triplet. ${\text{Pr}}(p_{i} |r_{j} )$ is the probability that the pattern $p_{i}$ correctly extracts the relation word $r_{j}$.

The conditional probability ${\text{Pr}}(p_{i} |r_{j} ){ }$ is calculated as given in Eq. (4),

4 $$\Pr \left( {p_{i} {|}r_{j} } \right) = \frac{{\Pr \left( {r_{j} {|}p_{i} } \right)\Pr \left( {p_{i} } \right)}}{{\Pr \left( {r_{j} } \right)}} = \frac{{\Pr \left( {r_{j} {|}p_{i} } \right)\Pr \left( {p_{i} } \right)}}{{\Pr \left( {r_{j} {|}p_{i} } \right)\Pr \left( {p_{i} } \right) + \Pr \left( {r_{j} {|}\neg p_{i} } \right)\Pr \left( {\neg p_{i} } \right)}}$$

where $\Pr \left( {p_{i} } \right)$ is the prior probability that $p_{i}$ is correct, which is equivalent to the precision of the pattern, and $\Pr \left( {r_{j} |p_{i} } \right)$ is the probability that the pattern $p_{i}$ extracts $r_{j}$ when $p_{i}$ is correct.

## Results

### Performance evaluation of relation detection and extraction

We first evaluated our model using the DDIE2013 data obtained from a previous study^47,48^. Because the label in this dataset is the absence or existence of the relationship between entities, we only evaluated the first part of our method, which is relation detection. The training set consisted of 4018 positive DDIs and 23,756 negative DDIs, and the test set consisted 979 positive DDIs and 4737 negative DDIs. We used the softmax function and Adam optimizer for binary classification and measured precision, recall, and F-score. In the training, the learning rate was 0.001, the training epoch was 30, the input layer dropout rate was 0.7, and the output layer dropout rate was 0.5. For the test set, we achieved a precision rate of 0.822, recall of 0.778, and F-score of 0.800 for the binary classification (Table 1). In comparison with the existing methods, our method showed better performance than most of the current machine learning-based methods except one. Compared to Tree-LSTM's Two-Stage Model, the F-score was lower, but precision was 1.6% higher.

**Table 1:** Performance evaluation for relation detection using DDIE2013 and dataset.

Model	Precision	Recall	F-score
SCNN's two-stage model^38^	77.5	76.9	77.2
Tree-LSTM's two-stage model^56^	80.6	84.2	81.8
pubmedBERT^25^	89.2	90.1	89.6
Our two-stage model	82.2	77.8	80.0

**Figure 5:**
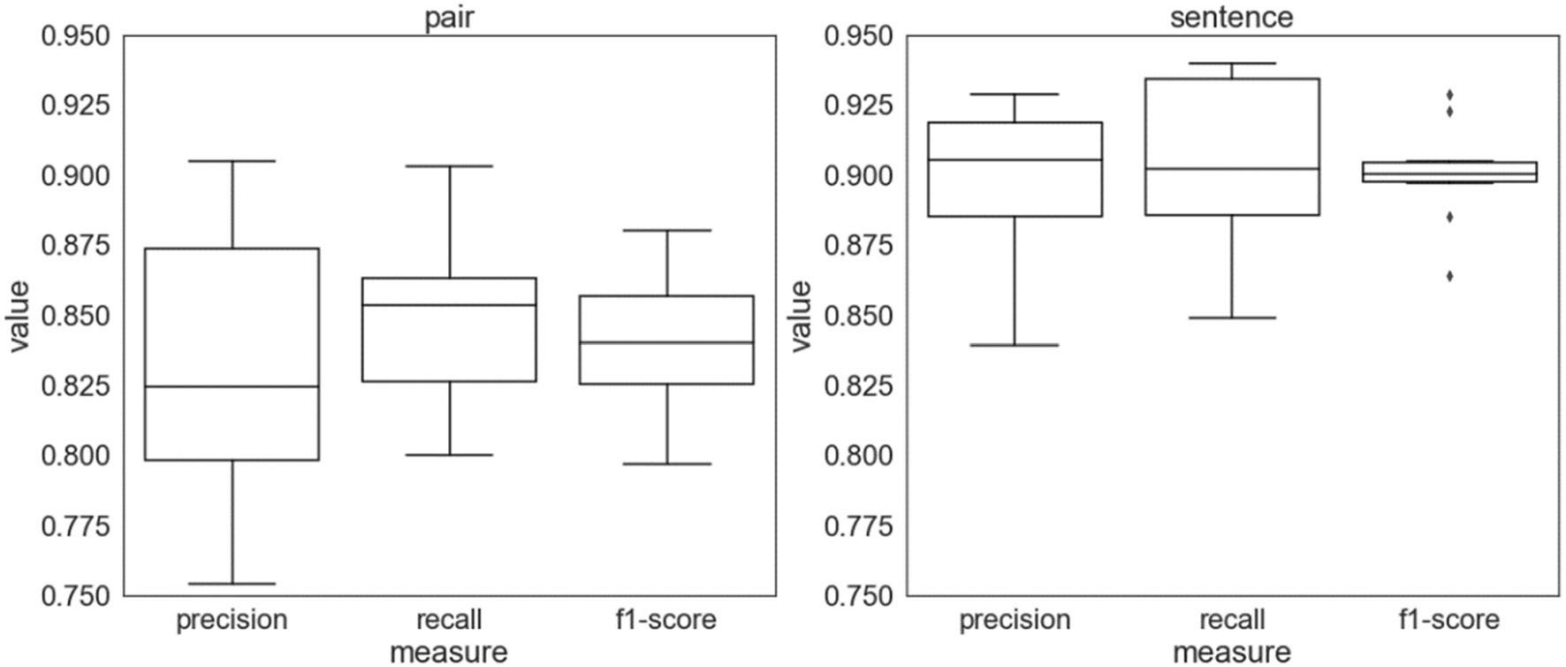
Performance of our method on bacteria-disease relation extraction. In tenfold cross validation, the mean precision was 0.832, the mean recall was 0.848, and the mean F-score was 0.839 for the pairs of bacteria and disease. For the sentences, mean precision was 0.898, the mean recall was 0.905, and the mean F-score was 0.901.

We also evaluated the entire model of both relation detection and extraction using an in-house evaluation dataset for microbe-disease interaction. Since the golden standard dataset is not available for microbe-disease interaction, we randomly selected 1000 sentences with 1269 positive relations and 572 negative relations from the abstracts downloaded from the PubMed repository (See ‘Method”). We performed tenfold cross-validation to improve the reliability of the evaluation. The sentences were split into 10 subsets of 100 sentences. The nine subsets were used as training data, and the remaining subset was used as validation data. The validation process was performed ten times, and each subset was used as validation data once. Finally, the results were averaged to calculate a single estimate, which resulted in a precision of 0.832, a recall of 0.848, and an F-score of 0.839 for all pairs of microbes and disease, on average (Fig. 5). When the accuracy was evaluated by sentence, it resulted in a precision of 0.898, a recall of 0.905, and an average F-score of 0.901, which is slightly higher than that of the entity pair.

Combined with relation detection, the relation extraction model was evaluated using macro-averaged precision, recall, and F-score as performance measures. To identify a good confidence threshold in the ensemble model, tenfold cross-validation was performed to evaluate the extraction accuracy for each of the 10 confidence thresholds (from 0.0 to 0.9 at intervals of 0.1). As shown in Supplementary Table S1, the F-score is the best when the confidence threshold is 0.5, which was used for further analysis. When comparing the performance of the three approaches, structural pattern matching, dependency-based extraction, and the ensemble with these two methods, the ensemble method shows higher F-scores than either method, which implies that the methods successfully complement each other in the ensemble model. Table 2 shows the performance for relation extraction with three approaches using two different confidence thresholds of 0.0 and 0.5.

**Table 2:** Performance evaluation for extracting relation words between microbe and disease entities.

Method	Confidence threshold = 0.0			Confidence threshold = 0.5		
	Precision	Recall	F-score	Precision	Recall	F-score
TPE	81.04	67.09	73.28	92.53	64.60	75.89
DBE	77.78	72.09	74.78	90.46	70.87	79.35
Ensemble	77.47	82.63	79.87	89.33	81.76	85.24

*TPE* structural pattern matching only, *DBE* dependency-based extraction only.

### Discovery of frequent associations between microbe and disease

**Figure 6:**
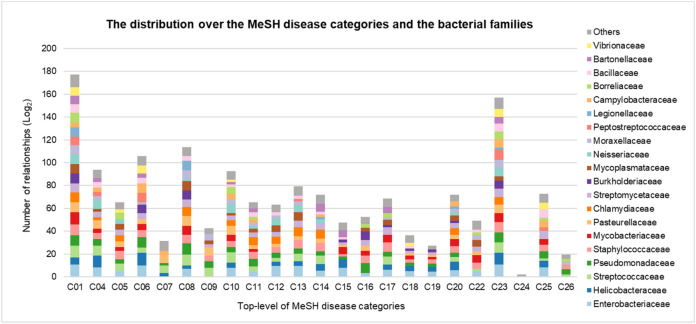
Distribution over the top-level MeSH disease categories and the bacterial families. The distribution of 30,085 relationships between 432 diseases and 319 bacteria is represented. The bacteria are shown in the top 20 bacterial families and others.

Our system was applied to analyze the microbe-disease association found in literature. Abstracts with the keyword ‘bacteria’ were collected from the Medline literature collection. After applying NER for microbe and disease names, 71,899 sentences were found to contain words related to disease and microbes, from which 52,251 sentences were predicted as sentences that describe microbe-disease association by our hierarchical LSTM classifier. Using the ensemble model, a total of 60,467 microbe-disease relations were extracted. To better analyze the association to the specific disease, the 14,306 association pairs related to the named entity 'infection' were excluded. For reliability, when the number of pairs for a specific microbe-disease association was below the average frequency (< 4), such an association was not included for further analysis. Finally, a total of 30,085 associations were retained, which were categorized based on the MeSH disease categories (Fig. 6).

Among 24 MeSH disease categories, ‘Infections [C01]’ is the category where the most abundant disease-microbe associations were found from the biomedical literature, followed by ‘Pathological Conditions, Signs and Symptoms [C23]’, ‘Digestive System Diseases [C06]’, ‘Respiratory Tract Diseases [C08]’, and ‘Neoplasms [C04]’ (Table 3). The five most frequent bacterial families were Enterobacteriaceae, Helicobacteraceae, Streptococcaceae, Mycobacteriaceae, and Staphylococcaceae, which constituted 16.52%, 13.11%, 8.13%, 6.51%, and 5.96% of bacteria-disease associations, respectively (Table 4).

**Table 3:** Number of microbe-disease relations clustered by MeSH disease categories.

Disease category	# of relations	# of microbes	# of diseases
Infections	12,250 (40.72%)	210	171
Pathological conditions, signs and symptoms	7540 (25.06%)	148	69
Digestive system diseases	5137 (17.07%)	77	50
Respiratory tract diseases	4209 (13.99%)	81	43
Neoplasms	2632 (8.75%)	62	26
Female urogenital diseases and pregnancy complications	1831 (6.09%)	63	34
Nervous system diseases	1632 (5.42%)	55	40
Cardiovascular diseases	1183 (3.93%)	60	24
Male urogenital diseases	1094 (3.64%)	35	23
Chemically-induced disorders	1046 (3.48%)	49	11

**Table 4:** Number of microbe-disease relations categorized by bacterial families.

Bacteria family	# of relations	# of bacteria	# of diseases
Enterobacteriaceae	4969 (16.52%)	17	139
Helicobacteraceae	3944 (13.11%)	7	90
Streptococcaceae	2446 (8.13%)	21	72
Mycobacteriaceae	1958 (6.51%)	15	48
Staphylococcaceae	1794 (5.96%)	8	77
Pseudomonadaceae	1669 (5.55%)	5	66
Pasteurellaceae	1194 (3.97%)	15	66
Chlamydiaceae	933 (3.1%)	6	48
Peptostreptococcaceae	682 (2.27%)	3	19
Streptomycetaceae	658 (2.19%)	2	50

In the Infections category [C01], a total of 12,250 relations were extracted, of which 210 bacteria and 171 diseases were involved. The most frequent disease was pneumonia, which also belongs to another category of respiratory tract diseases in MeSH. The species frequently associated with pneumonia were *Streptococcus pneumoniae* in 363 relations, *Pseudomonas aeruginosa* in 148, *Staphylococcus aureus* in 138, and *Mycoplasma pneumoniae* in 107. The other frequent diseases were tuberculosis, sepsis, and bacteremia, found in 1,050, 911, and 637 relations with 15, 47, and 38 bacteria, respectively.

**Figure 7:**
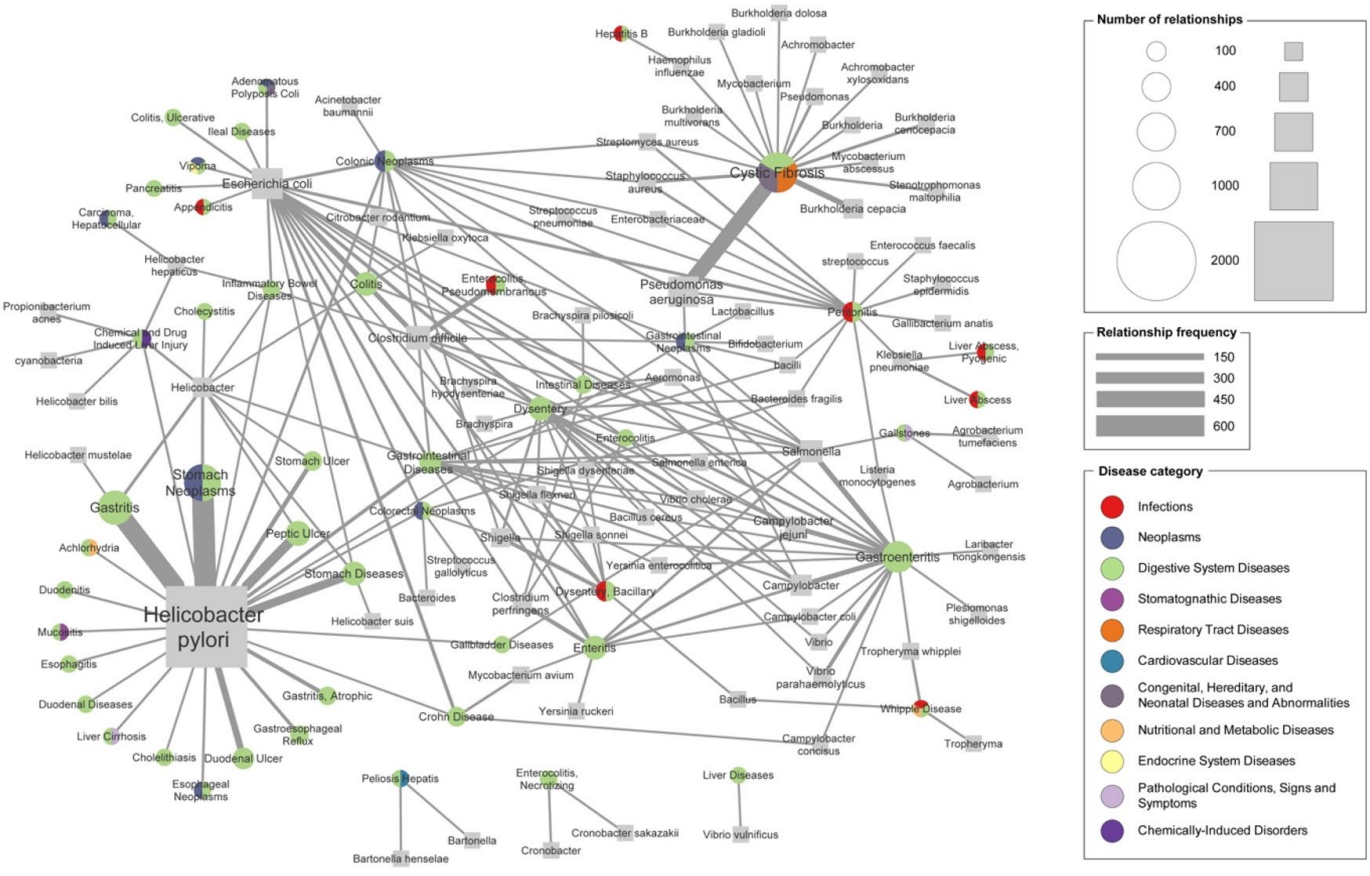
Network for digestive system diseases. A disease node (circle) and a microbe node (square) are connected by an edge when more than four relations are extracted. The size of node is proportional to the number of extracted relations between the disease or microbe, and the color of node represents top-level MeSH disease categories to which the disease belongs. The width of edge is proportional to the frequency at which the relation is extracted.

In Digestive System Diseases [C06], 5137 relations related to 77 bacteria and 50 diseases were extracted from the literature (Fig. 7). The disease with the most abundant microbes was cystic fibrosis, which is also associated with respiratory tract disease and genetic diseases in MeSH categories. Since its physiology is related to the pancreas and intestine in addition to lung infection^55^, diverse roles and effects of bacteria have been studied. Cystic fibrosis had 735 relationships with 16 bacteria. The other frequent diseases were stomach neoplasms, gastritis, and gastroenteritis with 650, 558, and 470 relations, respectively. The most frequent bacteria were *Helicobacter pylori* in 2081 relations, *Escherichia coli* in 463, *Pseudomonas aeruginosa* in 451, and *Clostridium difficile* in 244.

In respiratory tract diseases [C08], there were a total of 4209 relations that consisted of 43 diseases and 81 bacteria from 30 bacterial families. The two diseases highly associated with microbes were pneumonia and cystic fibrosis, which were the most abundant in infection and digestive disease categories, respectively. The other frequent diseases were lung diseases in 307 relationships and respiratory insufficiency in 246 relationships. The most frequent bacteria were *Streptococcus pneumoniae* in 502 relationships, *Pseudomonas aeruginosa* in 346, and *Haemophilus influenzae* in 243.

### Disease-disease relationship based on shared bacteria

**Figure 8:**
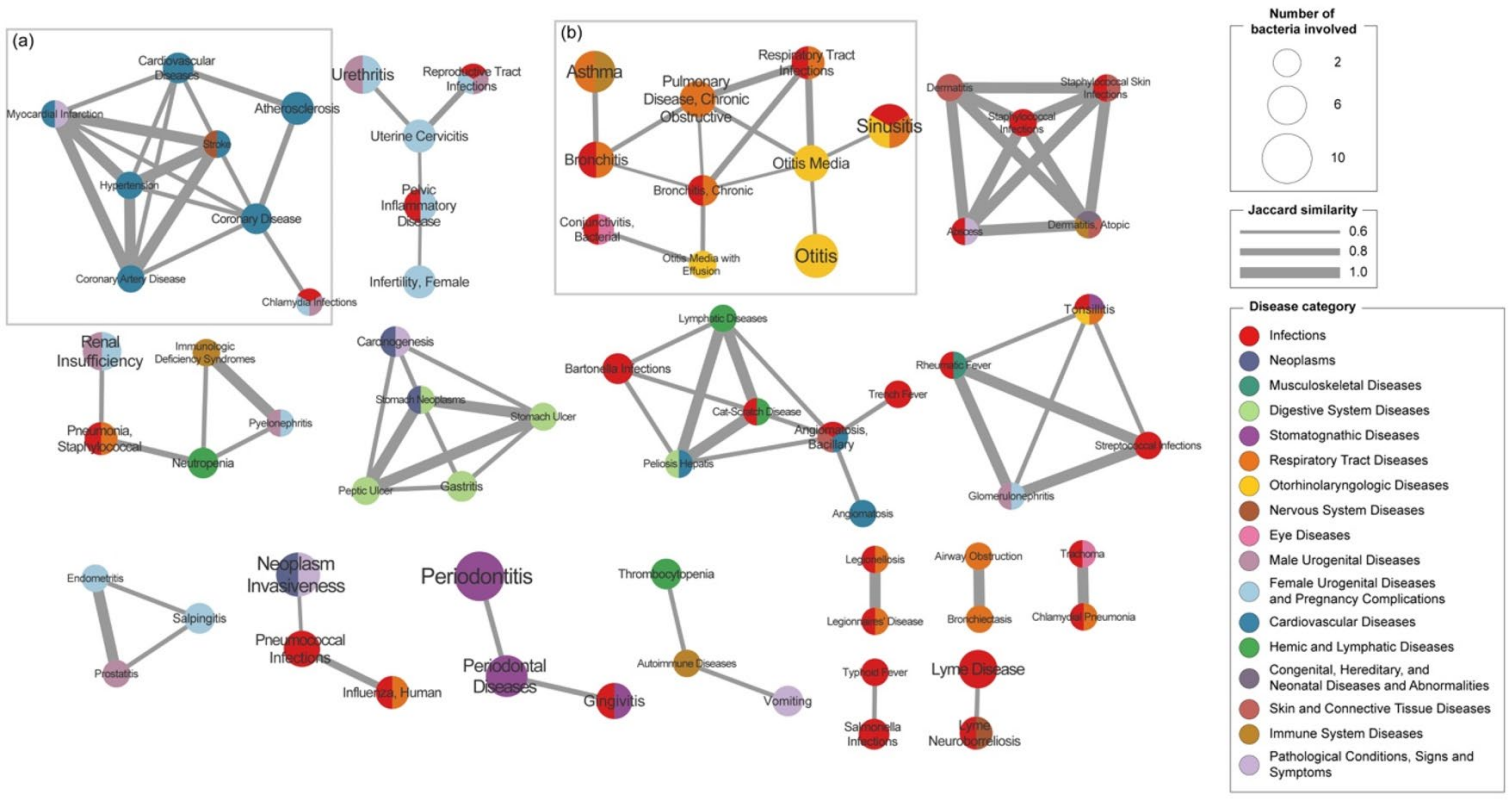
Disease similarity network. The network shows Jaccard similarities among diseases calculated with relevant microbes. The size of node is proportional to the number of microbes involved. The color of node represents top-level MeSH disease categories to which the disease belongs. The disease nodes are connected by an edge if Jaccard similarity was 60% or more. The width of edge is proportional to the similarity among nodes.

To investigate the similarity between diseases with respect to shared bacteria, a Jaccard index was applied. The higher the Jaccard index, higher the relation between the two diseases and the bacteria involved. For similarity calculation, the diseases associated with only one common bacterium were excluded, which can provide more reliable pairs of diseases with common bacteria. As a result, the similarity of 8958 pairs of diseases was calculated from the 230 diseases retained, ranging from 1 to 100%. Figure 8 shows a disease–disease network with a Jaccard index of 60% or more similar among diseases. The network consisted of 71 diseases, and 89 pairs of diseases shared more than 60% of microbes.

For a similarity network with a Jaccard index of 60% or more, the largest node is periodontitis related to ten bacteria, followed by sinusitis, otitis, and neoplasm invasiveness. Most diseases show a high Jaccard index for diseases in the same MeSH categories. In Fig. 8a, for example, all diseases except chlamydia infections belonged to cardiovascular diseases. All diseases of the sub-network were related to *Chlamydophila pneumoniae*, of which cardiovascular diseases were also related to *Helicobacter pylori*. Figure 8b shows the similarities between respiratory tract diseases and otorhinolaryngologic diseases. The diseases in the sub-network belonged to different categories, but all of them were associated with *Streptococcus pneumoniae* and *Haemophilus influenzae*. In particular, respiratory tract infections and otitis media showed a high Jaccard similarity of 80%, despite belonging to different categories. They shared relationships with four bacteria: *Streptococcus pneumoniae*, *Haemophilus influenzae*, *Moraxella catarrhalis*, and *Pseudomonas aeruginosa*.

## Conclusion

In this article, we introduced a process that combines natural language processing and machine learning methods to analyze relations between diseases and microbes. A hierarchical LSTM model with six layers was proposed to detect the existence of relationships between microbe and disease within sentences. In this process, the hierarchical LSTM model was used to determine the presence or absence of relationships in a sentence. For sentences that were determined to have relations, two different parsing methods extracted relation words. Both results were combined using an ensemble model based on Bayes' theorem. Our model not only detected the relationship between the diseases and microbes but also predicted the relation word between them. Evaluation of the results showed that our process achieved an F-score of 0.8764 and 0.8524 in binary decisions and extracting relation words, respectively. As a case study, we performed a large-scale analysis of the relationship between microbes and disease. Additionally, a set of common microbes shared by multiple diseases was identified in this study. This investigation could provide information on the major microbes that are found or studied for a specific disease. Several databases of microbe-disease association are currently available, which are based on the analysis of only a limited number of publications. Our method represents the first systematic approach to find microbe-disease relation from the scientific articles by using an entire process from named entity recognition to relation word extraction. This approach allows a large-scale analysis on microbe-disease association with detailed information described in the literature.

## Supplementary Information

Supplementary Information
