# Supplementary material for: Discovering microbe-disease associations from the literature using a hierarchical long short-term memory network and an ensemble parser model
Source: Sci Rep. 2021 Feb 24;11:4490. doi: 10.1038/s41598-021-83966-8 (PMC7904816; doi:10.1038/s41598-021-83966-8)
Supplement: Supplementary file 1 — Supplementary Information [file 41598_2021_83966_MOESM1_ESM.docx]

**Supplementary Tables for**

**Discovering microbe-disease associations from the literature using a hierarchical long short-term memory network and an ensemble parser model**

Yesol Park^1^, Joohong Lee^1^, Heesang Moon^1^, Yong Suk Choi^1, §^, Mina Rho^1,2, §^

^1^Department of Computer Science and Engineering, Hanyang University, Seoul, Korea,

^2^Department of Biomedical Informatics, Hanyang University, Seoul, Korea

Supplementary Table S1. Performance of ensemble approach over confidence threshold

| Confidence threshold | Precision | Recall | F-score |
| --- | --- | --- | --- |
| 0.0 | 77.47 | 82.63 | 79.87 |
| 0.1 | 79.81 | 82.63 | 81.07 |
| 0.2 | 82.23 | 82.44 | 82.22 |
| 0.3 | 84.4 | 82.25 | 83.22 |
| 0.4 | 88.48 | 82.07 | 85.01 |
| **0.5** | **89.33** | **81.76** | **85.24** |
| 0.6 | 89.52 | 80.96 | 84.88 |
| 0.7 | 90.15 | 80.4 | 84.88 |
| 0.8 | 90.59 | 78.22 | 83.79 |
| 0.9 | 92.38 | 70.68 | 79.89 |
|  | | | |

Supplementary Table S2. Performance of microbe-disease relation detection

| Model | Precision | Recall | F-score |
| --- | --- | --- | --- |
| pubmedBert | 89.34 | 92.37 | 90.79 |
| Our Two-Stage Model | 90.00 | 92.80 | 91.31 |

Supplementary Table S3. Performance of microbe-disease relation word extraction (confidence=0.5) by different epochs and batch sizes

| Epoch | Batch size | | | | |  |
| --- | --- | --- | --- | --- | --- | --- |
|  | 16 | 32 | 64 | 128 | 256 | 512 |
| 10 | 83.69 | 84.48 | 85.06 | 83.44 | 83.94 | 84.71 |
| 20 | 83.46 | 84.87 | 83.14 | 85.02 | 83.67 | 84.23 |
| 30 | 84.54 | 83.52 | 83.46 | 84.15 | 82.55 | 84.10 |
| 40 | 85.09 | 84.44 | 84.16 | 84.13 | 84.19 | 84.75 |
| 50 | 85.22 | 85.24 | 84.70 | 84.00 | 84.41 | 84.28 |
